# The chromosomal-scale genome sequencing and assembly of *Athetis lepigone*

**DOI:** 10.1038/s41597-024-03136-z

**Published:** 2024-04-05

**Authors:** Alexander Yesaya, Lei Zhang, Chao Wu, Yiheng Fu, Ji Zhang, Jingjie An, Yutao Xiao

**Affiliations:** 1 Shenzhen Branch, Guangdong Laboratory of Lingnan Modern Agriculture, Key Laboratory of Gene Editing Technologies (Hainan), Ministry of Agriculture and Rural Affairs, Agricultural Genomics Institute at Shenzhen, Chinese Academy of Agricultural Sciences, 518120 Shenzhen, China; 2 State Key Laboratory for Conservation and Utilization of Subtropical Agro-Bioresources, Guangxi University, 530005 Nanning, China; 3 Sanya Nanfan Research Institute and College of Tropical Crops, Hainan University, Sanya, 572025 China; 4 Plant Protection Institute, Hebei Academy of Agriculture and Forestry Sciences, Key Laboratory of Integrated Pest Management on Crops in Northern Region of North China, Ministry of Agriculture and Rural Affairs, IPM Innovation Canter of Hebei Province, International Science and Technology Joint Research Canter on IPM of Hebei Province, Baoding, China

**Keywords:** Agricultural genetics, Sequence annotation, DNA sequencing

## Abstract

*Athetis lepigone* is an emerging highly polyphagous insect pest reported to cause crop damage in several European and Asian countries. However, our understanding of its genetic adaptation mechanisms has been limited due to lack of high-quality genetic resources. In this study, we present a chromosomal-level genome of *A. lepigone*, representing the first species in the genus of *Athetis*. We employed PacBio long-read sequencing and Hi-C technologies to generate 612.49 Mb genome assembly which contains 42.43% repeat sequences with a scaffold N50 of 20.9 Mb. The contigs were successfully clustered into 31 chromosomal-size scaffolds with 37% GC content. BUSCO assessment revealed a genome completeness of 97.4% with 96.3 identified as core Arthropoda single copy orthologs. Among the 17,322 genes that were predicted, 15,965 genes were functionally annotated, representing a coverage of 92.17%. Furthermore, we revealed 106 P450, 37 GST, 27 UGT, and 74 COE gene families in the genome of *A. lepigone*. This genome provides a significant and invaluable genomic resource for further research across the entire genus of *Athetis*.

## Background & Summary

*Athetis lepigone* (Möschler, 1860) is an emerging outbreak insect pest that was originally recorded from South Sweden, South Finland and East Austria in eastward direction across the steppe belt of Asia to China and Japan^1,2^. Perhaps, due to strong flight capacity, global warming and climate change, it began to spread out across many European and Asian regions^3,4^. In the past subsequent years, the infestation of this species exponentially expanded, leading to extensive damage to summer maize crops in the Huang-Huai-Hai Rivers Plain in China over an area of about 2.2 million hectares in the year of 2011. This pest is highly polyphagous, such that, it has been reported to cause damage to more than 30 species of plants from 13 different plant families and it is now considered as significant pest of wheat, maize and other crops in several Eurasian regions^5,6^. It produces four different host-fed generations annually and the host plant preference of the first generation is mainly winter wheat, preferably, the germinating wheat kernels, suggesting possible possession of overwintering traits in their genetic makeup^5^. Lately, they feed on other summer crops such as peanut, soybean, and sweet potato although the population density is generally lower^7,8^.

Some studies were conducted that revealed a lack of population genetic structure, strong gene flow and presence of trinucleotide repeats that have frequent AAG motif^9,10^. Despite the rapid spread of *A. lepigone* across many regions and continued threats to many crop species, its genomic dynamics that footprint adaptation, evolution and origin are still not well understood due to limited genetic data about this insect and the whole genus of *Athetis*. Understanding how future populations of *A. lepigone* may respond to regular climate changes and different ecological habitats is of vital importance to uncover past, recent and future autographs of molecular adaptation and evolution in their genomes. Nevertheless, this can only be achieved with an availability of high-quality genomic data that can lay out a foundation for further studies.

This paper presents the first chromosomal-level genome assembly of *A. lepigone* and the first from the genus *Athetis* using long-read sequencing data and Hi-C sequencing technologies. The 612.49 Mb genome assembly length was generated with a scaffold N50 of 20.9 Mb being achieved and the contigs were successfully clustered into 31 chromosomal-sized scaffolds. The assembly completeness and integrity were assessed by Benchmarking Universal Single-Copy Ortholog (BUSCO) analysis, which revealed 97.4% completeness. 17,322 protein coding genes were predicted and 92.17% of the predicted genes were functionally annotated. The relationship of this moth with other Noctuidae moths was uncovered by performing a phylogeny analysis which revealed related orthologs and divergence times between *A. lepigone* and *A. ipsilon* estimated at 16.53 million years ago. Gene family analysis revealed 106 Cytochrome P450, 37 Glutathione S-transferase (GST), 27 UDP-glucuronosyltransferase (UGT) and 74 Cholinesterase (COE) gene families in its genome. This chromosomal-level genome will lay out a genetic map and milestone for further studies on this emerging polyphagous insect pest and other closely related species from the genus. These further studies will significantly and substantially contribute to the development of proper and sustainable management strategies of this insect pest.

## Methods

### Sampling and genomic material extraction

Sample were collected from Baoding city (38°51′03″ N 115°29′25″ E) situated at Qingyuan county of Hebei province in China, one of the regions where the pest is highly prevalent and infested. The insects were then domesticated with noctuid artificial diet^11,12^ in controlled laboratory setting, (27 ± 2 °C, 16 L: 8D and RH 60 ± 5%) at Hebei Academy of Agriculture and Forestry Sciences. Lately, adult months were fed on 10% honey solution after emergence. During breeding, we were isolating the larva to avoid unintended mating which may result in inbreeding. Controlled one-pair mating was employed to produce the first and second generations (F1 and F2), subsequently, sibling mating was consistently conducted to ensure high genetic homozygosity. This was done to establish inbred strains for subsequent genome sequencing, Hi-C sequencing and RNA-seq experiments. The genomic DNA (gDNA) was extracted from a male month using the Qiagen Genomic DNA kit (Cat. no. 13323, Qiagen). NanoDrop One UV-vis spectrophotometer (Thermo Fisher Scientific) and Qubit 3.0 Fluorometer (Invitrogen) were used for quality validation and quantification of the extracted gDNA in accordance to the manufacturer’s protocols respectively. To assist in gene annotation, RNA was extracted using the RNeasy Mini extraction kit (Qiagen) from the 3^rd^ instar, 5^th^ instar, pupa and the female moth, which originated from the same inbred strains used in gDNA extraction. Similar approaches used in DNA quality validation were applied to validate the integrity, purity and concentration of extracted RNA.

### Library construction and sequencing

About 0.5 μg of extracted gDNA was used as an input to generate a PCR-free Illumina library using the Truseq Nano DNA HT Sample preparation Kit (Illumina). Initially, the gDNA was sheared into 350-bp fragments, as insert size and sequenced in 150-bp paired end layout on the Illumina HiSeq 1000 platform, this generated short paired-end Illumina reads. With 5 μg sheared DNA from the same individual, ~20-kb SMRTbell insert libraries were prepared and then sequenced on PacBio Sequel II system. A Single Molecule Real-Time (SMRT) bell express template prep kit 1.0 was used to produce continuous long reads (CLR) (Table 1). RNA sequencing was done using the extracted RNA from different samples which were used to build cDNA libraries using the NEBNext Ultra RNA library prep kit for Illumina^13^, we followed approaches as previously described^14^. Thereafter, the constructed cDNA libraries were sequenced on the Illumina NovaSeq 6000 platform following a paired end 150 bp layout, generating RNA-seq reads (Table 1). A male pupa from the same inbred strains was chosen for Hi-C library construction. To prepare the library, nuclear DNA was cross-linked *in situ*, extracted and digested using Mbol (GATC) restriction enzyme. Subsequently, Hi-C libraries were then amplified by 12–14 cycles of PCR before being sequenced on Illumina NovaSeq 6000 platform with a 150 bp paired-end set up to produce Hi-C reads data (Table 1).

**Table 1:** Statistical characteristics of the sequencing reads.

Sample name	Sequencing platform	Number of reads	Number of bases	GC content	Coverage
Alep_PacBio	PacBio sequel II	8,837,681	158.38 Gb	37.64	258X
Alep_Hi-C	Illumina NovaSeq 6000	221,466,097	64.2 Gb	38.38	105X
Alep_Illumina	Illumina HiSeq 1000	171,482,285	51.44 Gb	36.85	84X
RNA seq of larvae 5^th^	Illumina NovaSeq 6000	59,691,745	16.89 Gb	45.37	\
RNA seq of larvae 3^rd^	Illumina NovaSeq 6000	63,052,827	17.83 Gb	45.04	\
RNA seq of female	Illumina NovaSeq 6000	61,410,889	17.37 Gb	45.09	\
RNA seq of pupa	Illumina NovaSeq 6000	57,941,892	16.39 Gb	44.29	\

### Genome estimation and contig assembly

Genome examination is of critical importance in order to assess the main features, including, heterozygosity, genome size and repetitive sequence content prior to actual genome assembly. The k-mer distribution of 17 k-mer frequencies were generated from quality controlled short Illumina reads which were used as input to construct k- values using jellyfish^15^ and timer frequencies by jellyfish assessed the distribution of k-mers. The constructed k-mer frequencies were used to perform genome evaluation using Genome Scope v1^16^. Subsequently, genome size was estimated as 562.96 Mb with a heterozygosity of 2.31% and estimated unique sequences constituting to 47.2% (Table 2). The generated long reads from PacBio sequencing were assembled into contigs using Canu v2.1 software^17^. Primarily, PURGE_DUPS v1.2.3 was used in processing the contigs to filter out any heterozygous sequences following the default parameters (https://github.com/dfguan/purge_dups). The correction of assembly discrepancies was performed by Finisher_SC v2.1^18^. Primarily, quality validation of short Illumina raw reads was performed by trimming the adaptors using clean adapter v1.1 and the low-quality regions were polished by clean-lowqual v1.0, following the previously described methods^14,19^. Thereafter, BWA-MEM v0.5.7a-r405^20^, was used to align the filtered and cleaned short Illumina reads to the assembled contigs and then correction of single base errors in contigs was performed by pilon v1.23^21^.

**Table 2:** Analytical summary of genome assembly and genome estimation analysis.

Statistical feature	Corresponding value
Genome length	612.49 Mb
Scaffold N50	20.9 Mb
GC content	37%
Repeat sequence length	259.9 Mb
Estimated genome size	562.96 Mb
Estimated unique sequences	47.2%
Estimated Heterozygosity	2.31%
Estimated duplicated sequences	1.12%
BUSCO complete genes(C)	97.4%
BUSCO single copy genes(S)	96.3%
BUSCO duplicated genes(D)	1.1%
BUSCO fragmented genes(F)	0.6%
BUSCO missing genes (M)	2%

### Chromosomal-level assembly

Hi-C data was used to assign the draft scaffolds into chromosome-length scaffolds by employing 3D-DNA pipeline tools to detect chromosome interactions in the assembled draft scaffolds^22^. Initially, the Hi-C sequenced low quality reads (<20 bp and >30 bp reads) were removed using Fastp tool v0.20.0 and these filtered HiC reads data were then aligned to the assembled contigs using Bowtie2 software^23^. HiC-Pro tool was then used to generate intra and inter-chromosomal contact maps depicting interplays between genomic sites while removing unreliable data^24^, resulting in an initial scaffold-level assembly.

**Fig. 1:**
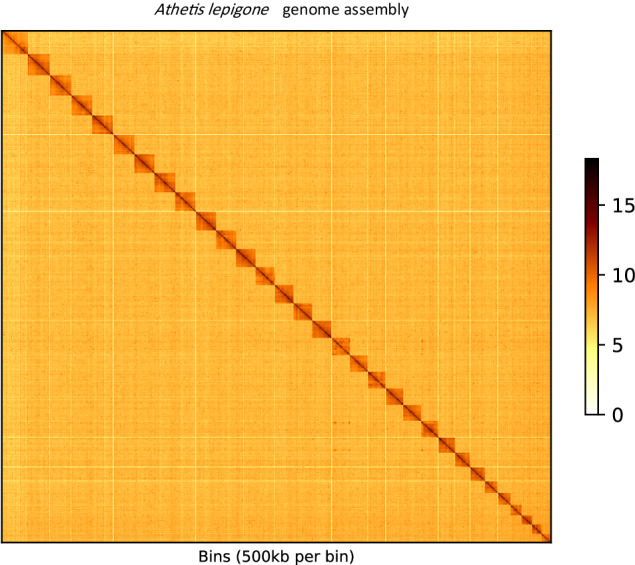
A heatmap matrix of *A. lepigone* generated from genome-wide HiC-data. It illustrates the interaction of 31 pseudo-chromosomes in the genome, as depicted by interlinked box-patches along the diagonal line, and the intensity of interaction conveyed through a colour band-scale.

The scaffold-level assembly was then processed with 3D-DNA by employing an agglomerative hierarchical grouping approach to cluster, orient and order scaffolds into chromosomal-length scaffold clusters hence generating 31 chromosomal-length scaffolds also referred as pseudo-chromosomes^22^. These clustered scaffolds were subjected to Juicer software^25^, to produce an interaction graph, reflecting interconnectedness between the clustered scaffolds with bin size resolution of 500 kb (Fig. 1). Afterward, a careful manual inspection and correction of any visual errors in the graph was done using JuiceBox^26^, generating an assembly that exhibits higher completeness and contiguity than the initial scaffold-level assembly. We finally obtained chromosomal-level genome of 612.49 Mb in size which was not very far from an estimated genome of 562.96 Mb. The resulting scaffold N50 length was 20.9 Mb and GC content in *A. lepigone* genome was observed to be 37%. The final chromosome interaction matrix was envisioned as a heatmap in the form of diagonal patches that are strongly linked based on the interplay signals between valid mapped reads and bins using JuiceBox tool (Fig. 1). Benchmarking Universal Single-Copy Ortholog (BUSCO) v4^27^, was employed to assess the integrity, purity and completeness of the genome using Arthropoda gene set (odb10). Out of the 1367 BUSCOs, 1332 BUSCOs were identified as complete, representing 97.4%, 15 BUSCO genes were identified as duplicates, 8 being fragmented and 27 identified as missing BUSCO genes (Table 2).

### Repeat elements prediction

**Fig. 2:**
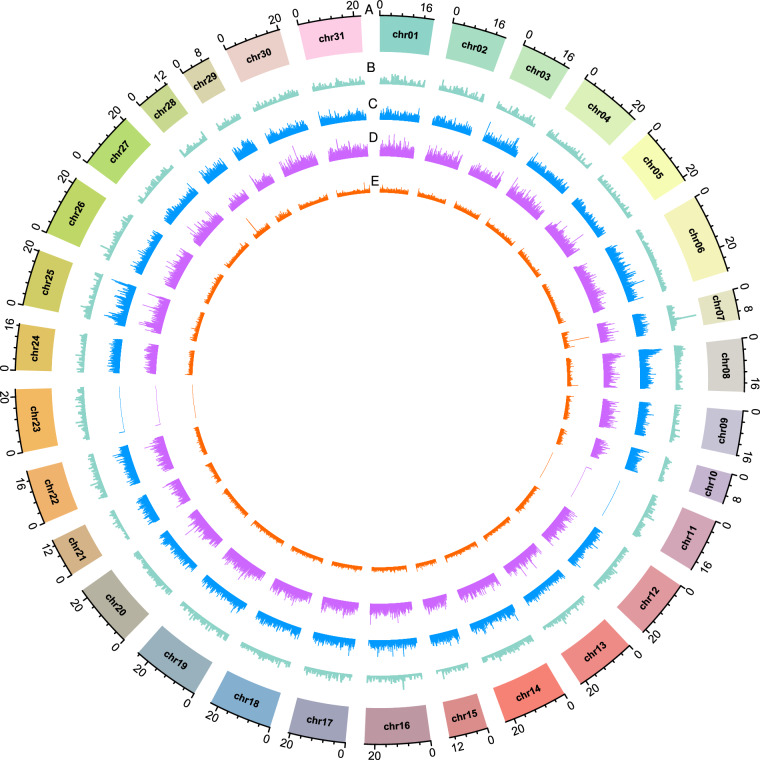
A circular visualization of chromosomes in *A. lepigone* genome. The outermost plot represents ideograms of 31 chromosomes (**a**). Moving from the second outermost track to the innermost track, each concentric circle denotes the density of genes (**b**), DNA transposons (**c**), LTR transposons (**d**) and, simple repeats (**e**).

Repeat elements identification from the assembled genome was done by firstly, generating a de novo repeat library using RepeatModeler v1.0.11 following default parameters^28^. RepeatMasker v4.07 (https://www.repeatmasker.org/), was then used to classify repeat families and search the de novo repeat library against the Repbase^29^, generating a final repeat sequence library. RepeatMasker was further used to predict the repeat elements from repeat sequence library based on Repbase library^29^. After completing the aforementioned analysis, we identified a total of 259.9 Mb as repeat sequence length representing 42.43% of the entire genome where the DNA transposons constituted to 3.85% and the long tandem repeats (LTRs) contributed to 3.56% (Table 3, Fig. 2).

**Table 3:** Statistical summary of repeat elements in the genome of *A. lepigone*, where column for others represent the total RCs, PLEs satellites, retroposons and low complexity elements.

Genomic features count	DNA transposons	LINEs	SINEs	LTRs	Simple repeats	Others	Unknown
Elements	78,603	161,856	100,848	56,899	103,158	254,194	480,310
Length (bp)	23,565,282	55,159,369	16,767,794	21,795,072	10,802,833	41,998,051	89,810,321
Percentage%	3.85	9	2.74	3.56	1.76	6.85	14.66
Total %	42.43

### Gene prediction and functional annotation

A multi-approach criterion for gene prediction and annotation in *A. lepigone* genome was performed by employing transcriptome-based prediction, ab initio prediction, and homolog-based gene prediction. Applying the default settings, de novo gene models were predicted using Ab initio gene prediction approach using AUGUSTUS v3.2.2^30^. However, the assembled genome was hard and soft-masked by RepeatMasker before performing gene prediction. We trained all gene prediction models from a set of proteins sequences generated from the RNA-Seq dataset (transcripts). Homology-based annotation was performed by searching the genome sequences containing non-intersecting protein sequences from related species. We employed geta v2.4.2 software to query the sequences (https://github.com/chenlianfu/geta). The queried genomes from the NCBI database included sequences from *Agrotis ipsilon* (GCA_028554685.1)^31^, *Agrotis segetum* (GCA_036375495.1), *Bombyx mori* (GCF_000151625.1)^32^, *Drosophila melanogaster* (GCF_000001215.4)^33^, *Helicoverpa armigera* (GCF_002156985.1)^34^, *Spodoptera frugiperda* (GCA_012979215.2)^35^, *Trichoplusia ni* (GCF_003590095.1)^36^, *Plutella xylostella* (GCF_000330985.1)^37^, *Manduca sexta* (GCF_014839805.1)^38^ and *Spodoptera littoralis* (GCA_022664705.1)^39^.

For the RNA-seq annotation, the quality controlled RNA-seq reads (by clean-lowqual v1.0) were aligned to the assembled genome of *A. lepigone* using TopHat2 v2.012^40^ and got processed using Cufflinks v2.2.1 tool^41^, applying default settings to produce transcript predictions. Applying the default parameters, Evidence Modeler version v1.1.1^42^, was used to integrate the three gene prediction models into an unified and consolidated gene dataset. The integrated unified dataset led to the prediction of 17,322 protein coding genes distributed across the genome and a mean gene length was found to be 14,029.25 bp. Gene functional annotation was executed by aligning the predicted protein sequences against the NCBI non redundant, Trembl, eggNOG, and KEGG databases by BLAST v2.3.0+ with E-value cut-off of <10-5, This resulted into 15,965 being functionally annotated genes representing 92.17% of the anticipated protein coding genes (Table 4). Using Lingbo MicroClass an online tool, we lately visualized some genomic feature distribution such as density of gene, DNA transposons, LTR transposons and simple repeats across the 31 clustered chromosomes of *A. lepigone* (Fig. 2) (http://www.cloud.biomicroclass.com/CloudPlatform/SoftPage/CIR).

**Table 4:** The analytical summary of gene functional annotation based on four different databases.

Annotation database	Predicated genes	Annotated genes	Percentage per platform
KEGG	17,322	12,674	73.17%
eggNOG	17,322	15,667	90.45%
Trembl	17,322	15,837	91.43%
NCBI-nr	17,322	15,806	91.25%
Total annotated genes		15,965	92.17%

### Comparative collinearity analysis

**Fig. 3:**
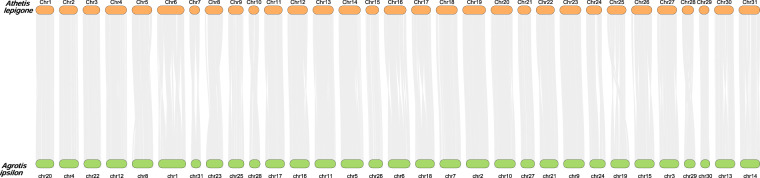
A syntenic relationship. The analysis reveals intricate relationships between *A. lepigone* and *A. ipsilon* chromosomes in their genomes.

Collinearity analysis was performed by investigating the genomic organization between *A. lepigone* (this study) and *A. ipsilon* (accession number GCA_028554685.1 on NCBI database), through a synteny analysis to assess collinear relationships in their chromosomes. Initially, we aligned their protein sequences using BLAST v2.226+, with a stringent E-value cutoff of less than 1E−10. Subsequently, we constructed syntenic blocks using the MCScanX software^43^, applying default parameters. Visualisation of the syntenic blocks was done by TBtools-II v2.008^44^. Our analysis revealed no evidence of fission between the two species, suggesting that structural continuity and integrity of chromosomes in both species have been predominantly preserved. Nevertheless, a noteworthy chromosomal rearrangement during the evolution of these species was observed (Fig. 3).

## Data Records

PacBio, Illumina and Hi-C sequencing data have been deposited to the NCBI Sequence Read Archive with accession numbers SRR26381158^45^, SRR263811156^46^ and SRR26381157^47^ respectively. Additionally, RNA-Seq data are available and active in the NCBI database with accession numbers SRX22293591^48^, SRX22293592^49^, SRX22293593^50^ and SRX22293595^51^. The assembled genome can be found on NCBI’s GenBank through accession number GCA_033675125.1^52^. Furthermore, for broader accessibility, we have deposited the assembled genome, gene annotation and repeat annotation data in the figshare database^53^.

## Technical Validation

### Quality assessment of the genomic material

After the genomic material extraction, we performed a thorough quality assessment to evaluate the purity, concentration, and integrity of both DNA and RNA using NanoDrop One UV-Vis spectrophotometer (Thermo Fisher Scientific) and a Qubit 3.0 Fluorometer (Invitrogen), ensuring that we have a high-quality genomic material.

### Genome assembly assessment

We employed Benchmarking Universal Single-Copy Orthologs (BUSCO) v4 to evaluate the genome assembly’s robustness and completeness. This assessment, using the Arthropoda gene set (odb10) database, revealed that 97.4% of the genes were present in the assembled genome. This suggests that a substantial majority of the essential and conserved genes were successfully captured, underscoring its high level of robustness and completeness (Table 2).

### Chromosomal clustering assessment

The integrity of chromosomal clustering was evaluated by examining the interactive intensity of contact heatmaps with 500 kb bin window size. Manual curation to correct assembly discrepancies performed by JuiceBox also improved our assembly. The Hi-C heatmap matrix clearly illustrated that there was a significantly strong intensity of interaction along the diagonal line of the heatmap plot, depicting the 31 distinct chromosomes. This clear pattern affirms the successful clustering of chromosomal-length scaffolds (Fig. 1).

### Assessing the validity of gene prediction and annotation

We adopted three different approaches to assess the quality and robustness of our gene prediction and annotation. Firstly, a BUSCO analysis of protein coding genes using Insecta_odb10 database, revealed a 93.5% completeness, comprising 91.4% as single copy, 2.1% duplicated, 1.0% fragmented and 5.5% missing BUSCOs.

**Fig. 4:**
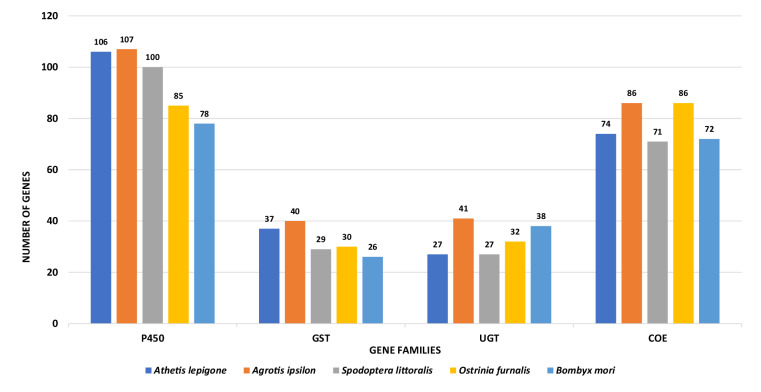
Comparative distribution of four gene families. The vertical axis indicates the number of genes whilst the horizontal axis indicates the corresponding gene families in *A. lepigone* genome and other lepidopteran species, suggesting a successful gene prediction and annotation.

Secondly, we analysed the presence of some gene families by accessing the protein sequences from the NCBI GeneBank which were subjected to manual curation to generate pure reference protein sequences for each gene family. Then, BLAST+ (BLAST v2.3.0+) (E-value < 1E-5) was employed to identify the potential gene sequences in *A. lepigone* by contrasting with purified reference sequences. The identified potential genes were further scrutinized by HMMER v3 search^54^, with a cutoff E-value of <1E-5 by applying the Pfam database to validate the preserved regions in each gene family^55^. The clustering of some predicted genes into known gene families such as GST, P450, UGT and COE, indicates the presence of functionally conserved genomic regions in the sequence hence supporting the success of our gene prediction and annotation analysis (Fig. 4). Subsequently, we utilized an online tool to located and map the identified gene families across the chromosomes^56^ (Supplementary).

**Fig. 5:**
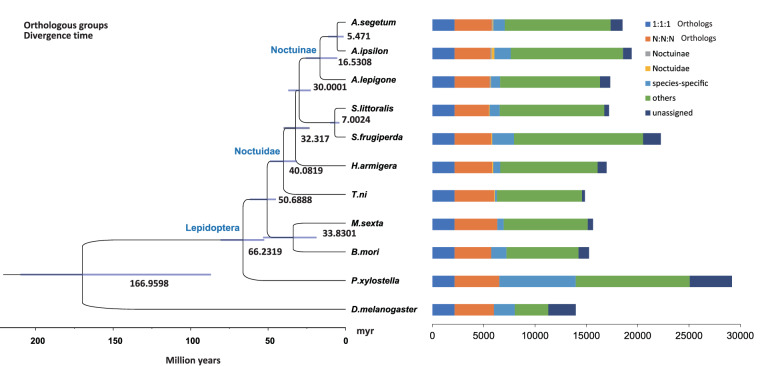
The species tree, providing estimates of divergence times for *A. lepigone* compared to other insect species. The figure also includes the distribution of orthologous groups across the genomes of 11 insect species, where 1:1:1 signifies single-copy orthologs and N: N: N represents orthologs with variable copy numbers.

Lastly, we assessed the orthologs in *A. lepigone* by analysing its protein sequence against the sequences of insect species used in gene prediction and annotation. This was processed by OrthoFinder v2.4.0^57^ with default settings. This primarily involved applying DIAMOND software^58^ for sequence alignment and employing the Markov Cluster Algorithm for orthogroup grouping^59^. Consequently, we aligned single-copy orthologous sequences from the species using MUSCLE v3.8.31^60^. Thereafter, a species tree, based on orthologs was constructed by employing Random Accelerated Maximum Likelihood-Next Generation (RAxML-NG) v1.0.2^61^, using the optimal method with 1000 bootstraps. Fig-Tree v1.4.4 an online tool was used for visualization (http://tree.bio.ed.ac.uk/software/figtree/) (Fig. 5). The clustering of predicted genes into known orthologs such as single copy orthologs, variable copy orthologs, Noctuinae and Noctuidae orthologs, provide evidence for the accuracy and quality of our gene prediction.

### Supplementary information

Supplimentary figure

## Data Availability

No custom codes were used in this study. All bioinformatics tools and software applications were executed in accordance with their respective manuals and protocols. The specific software versions and the parameters used are detailed in the methods section.
