# Supplementary material for: The chromosomal-scale genome sequencing and assembly of Athetis lepigone
Source: Sci Data. 2024 Apr 5;11:338. doi: 10.1038/s41597-024-03136-z (PMC10997617; doi:10.1038/s41597-024-03136-z)
Supplement: Supplementary file 1 — Supplimentary figure [file 41597_2024_3136_MOESM1_ESM.pdf]

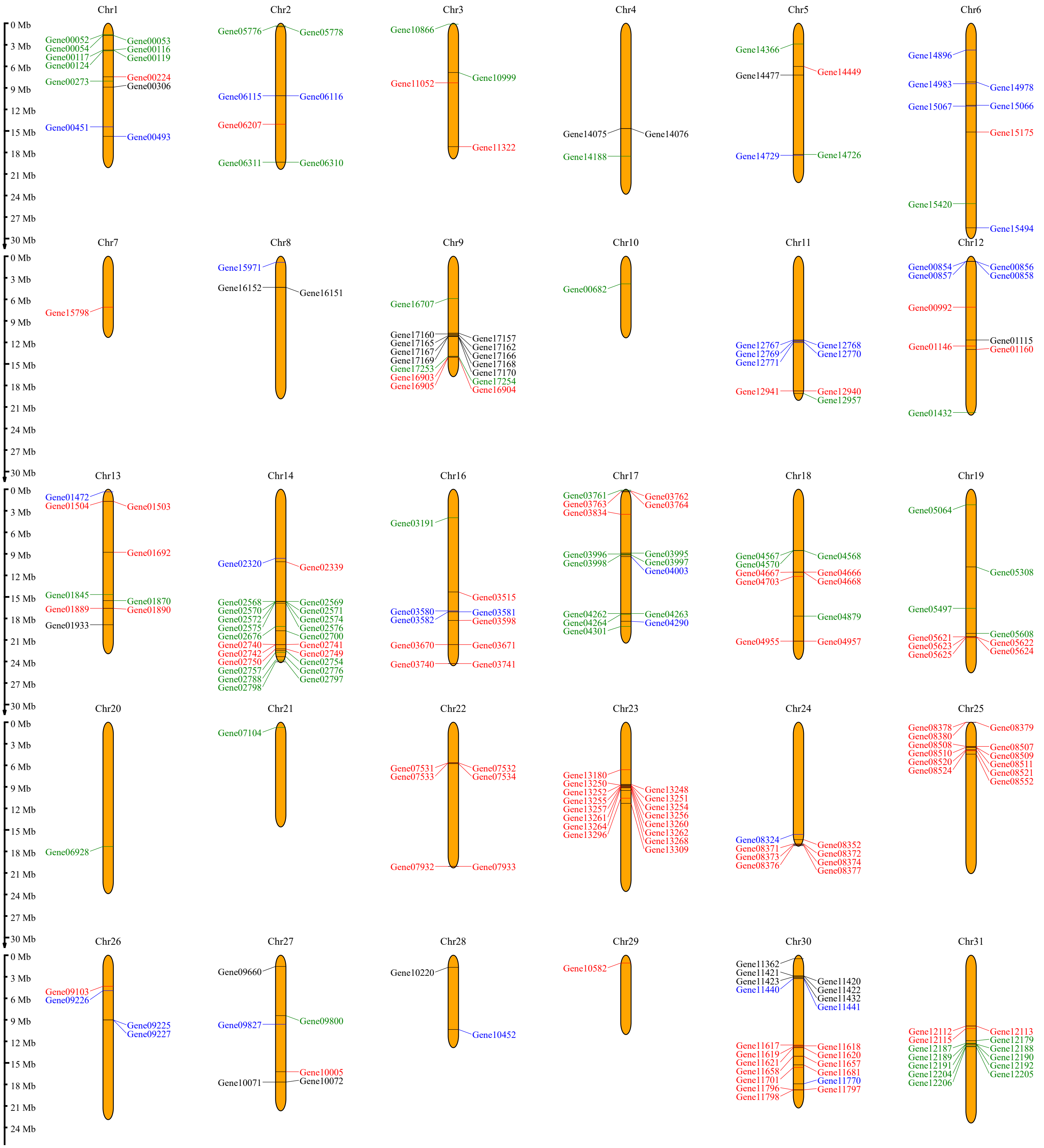

Fig. S1: Illustrate chromosomal distribution of key gene families with P450 genes marked in red, GST genes in blue, COE genes in green, and UGT genes in black, conveyed through their corresponding gene identity numbers.
